# Design of Bio-Responsive Hyaluronic Acid–Doxorubicin Conjugates for the Local Treatment of Glioblastoma

**DOI:** 10.3390/pharmaceutics14010124

**Published:** 2022-01-05

**Authors:** Alessio Malfanti, Giuseppina Catania, Quentin Degros, Mingchao Wang, Mathilde Bausart, Véronique Préat

**Affiliations:** Advanced Drug Delivery and Biomaterials, Louvain Drug Research Institute, University of Louvain, 1200 Brussels, Belgium; giuseppina.catania@studenti.unipd.it (G.C.); quentin.degros@student.vinci.be (Q.D.); mingchao.wang@uclouvain.be (M.W.); mathilde.bausart@uclouvain.be (M.B.)

**Keywords:** glioblastoma, local treatment, hyaluronic acid, polymer–drug conjugates, bio-responsive linkers

## Abstract

Glioblastoma is an unmet clinical need. Local treatment strategies offer advantages, such as the possibility to bypass the blood–brain barrier, achieving high drug concentrations at the glioblastoma site, and consequently reducing systemic toxicity. In this study, we evaluated the feasibility of using hyaluronic acid (HA) for the local treatment of glioblastoma. HA was conjugated to doxorubicin (DOX) with distinct bio-responsive linkers (direct amide conjugation HA-NH-DOX), direct hydrazone conjugation (HA-Hz-DOX), and adipic hydrazone (HA-AdpHz-DOX). All HA-DOX conjugates displayed a small size (less than 30 nm), suitable for brain diffusion. HA-Hz-DOX showed the best performance in killing GBM cells in both 2D and 3D in vitro models and displayed superior activity in a subcutaneous GL261 tumor model in vivo compared to free DOX and other HA-DOX conjugates. Altogether, these results demonstrate the feasibility of HA as a polymeric platform for the local treatment of glioblastoma and the importance of rationally designing conjugates.

## 1. Introduction

Glioblastoma (GBM) is the most common malignant primary central nervous system tumor in adults, representing approximately 57% of all gliomas and 48% of all primary brain cancers [1]. Furthermore, the associated morbidity, with a progressive decline in neurologic function and quality of life, can have a devastating impact on patients, caregivers, and families [2]. The current standard of care to tackle GBM has remained almost unchanged since 2005 and consists of a multimodal approach that involves surgery, radiotherapy, and systemic therapy (temozolomide). Despite recent advances in drug discovery and delivery, the overall prognosis remains poor, and long-term survival is rare, making GBM a significant unmet clinical need [3,4]. The minimal improvement in outcomes for GBM patients is due to several factors, including (1) the high propensity of GBM to migrate and invade surrounding healthy brain parenchyma; (2) GBM is located in the brain, which is shielded by the blood–brain barrier (BBB), the major obstacle for the systemic delivery of drugs in the brain, inhibiting the efficacy of the majority of treatments; (3) intrinsic resistance to current therapies due to high inter- and intra-patient heterogenicity, making it difficult to target and efficiently treat the entire GBM [5].

Localized strategies for delivering therapeutics and nanomedicines to the GBM (including intratumoral injection, convention-enhanced delivery, and filling the resection cavity) are an exciting approach that allows us to circumvent the BBB and achieve high drug doses in the brain while minimizing both local and systemic toxicity in order to overcome tumor resistance [6,7,8]. An example of a local treatment is Gliadel, a wafer loaded with carmustine, locally implanted in the brain after tumor resection, that was approved by the U.S. Food and Drug Administration in 1996 for the treatment of recurrent GBM [9]. However, the clinical practice showed that, together with a modest increase in survival, Gliadel has a short drug release period and can induce serious side effects.

Nanomedicine and therapeutics must be able to penetrate within the brain microenvironment to reach infiltrative cells that cause tumor recurrence and to provide more uniform drug distribution within tumors [10,11]. Therefore, in the last several years, many works have focused their attention on exploring new polymeric nanomedicines (using polymer nanoparticles, hydrogels, fibers, sprays) that could bypass Gliadel’s drawbacks and allow therapeutics’ diffusion in the GBM by active and passive targeting, achieving high local doses and improving survival [12]. Hanes and colleagues developed paclitaxel (PTX)-loaded, poly(lactic-*co*-glycolic acid) (PLGA)-*co*-PEG block copolymer nanoparticles with an average diameter of 70 nm, showing brain-penetrating efficacy and thus leading to improved paclitaxel efficacy in malignant glioma following local administration [13,14]. Our group designed a photopolymerizable polymer-based hydrogel for the local delivery of temozolomide and paclitaxel after the postsurgical treatment of GBM that resulted in long-term survivors (80%) in orthotopic U87-MG-bearing mice [15]. DOX-loaded acetylated dextran nanofibrous scaffolds were designed with different degradability rates, leading to complete remission in 43% of mice orthotopically challenged with U87-MG cells [16]. Rahman and colleagues engineered a hydrogel composed of low-methoxyl pectin encapsulating etoposide or olaparib drug nanocrystals coated with polylactic acid–polyethylene glycol; delivery as a spray showed deeper brain penetration [17]. However, despite the recent interesting achievements, most of the nanocarriers described so far suffer from poor translation to the clinic due to low biodegradability, potential neurotoxicity, poor drug loading, and inadequate drug release kinetics, which prevent the drug from reaching the peripheral GBM [6,18].

To address this challenge, we engineered hyaluronic acid (HA) with doxorubicin (DOX). HA is an excellent candidate to deliver drugs due to its biocompatibility, hydrophilicity, and multivalency, offering the possibility to introduce bio-responsive moieties on its backbone, allowing a stimuli-responsive therapeutic release [19]. HA has been extensively used to coat various nanocarriers, taking advantage of its intrinsic targeting towards CD44-overexpressed tumors, including GBM [20,21,22]. HA has been conjugated to therapeutics, such as paclitaxel [23,24], doxorubicin [25,26], and cisplatin [27], either alone or in combination [28,29], to improve drug efficacy and reduce toxicity. HA can act as a trojan horse to promote the cell uptake of the conjugated drug. To the best of our knowledge, HA-drug conjugates have shown efficacy in several cancer xenografts through both subcutaneous [30] and intravenous administration [31], but no report in the context of HA-drug conjugates for the local treatment of GBM has been published. Hence, the selected anticancer drug DOX was conjugated to HA to provide a high local concentration and more sustained release than free DOX when locally delivered for GBM treatment.

In this “proof-of-concept” study, we used an optimized and scalable synthetic procedure to develop a rationally designed family of HA-DOX conjugates suitable for the local treatment of GBM. Different bio-responsive linkers were studied to achieve the best therapeutic outcome. The novel HA constructs demonstrated enhanced cytotoxicity in vitro compared controls on different 2- and 3D spheroid GBM cell lines. The anti-tumor efficacy was also evaluated in vivo, on a subcutaneous syngeneic GBM in tumor-bearing mice, showing the impact of the linking chemistry of the conjugates on the therapeutic outcome.

## 2. Materials and Methods

### 2.1. Materials and Reagents

Hyaluronic acid (HA, MW = 100 kDa) was purchased from Lifecore Biomedical, LLC (Chaska, MN, USA). Doxorubicin (DOX) hydrochloride was obtained from Chemieliva Pharmaceutical Co., Ltd. (Chongqing, China). Tris(4-(dimethylamino) phenyl) methylium chloride (crystal violet), ethylenediaminetetraacetic acid (EDTA), paraformaldehyde (PFA), 4,6-diamidino-2-phenylindole (DAPI), and Alamar blue were purchased from Thermo Fisher Scientific (Waltham, CA, USA). Hydrazine, adipic dihydrazide, 4-(4,6-Dimethoxy-1,3,5-triazin-2-yl)-4-methylmorpholinium chloride (DMTMM Cl), bovine serum albumin (BSA), methyl cellulose (4000 cP), and Triton 100-X were purchased from Sigma-Aldrich (St. Louis, MO, USA). Cell culture media and reagents were obtained from Gibco (ThermoFisher Scientific, Waltham, CA, USA). Anti-LAMP-1 (lysosomal-associated membrane protein 1) was purchased from Miltenyi Biotec (Bergisch Gladbach, Germany), and FITC anti-mouse/human CD44 antibody was purchased from Biolegend (San Diego, CA, USA). If not specified, all the reagents were purchased from Sigma-Aldrich (St. Louis, MO, USA).

### 2.2. Synthesis and Characterization of HA-DOX Conjugates

#### 2.2.1. Synthesis of HA-NH-DOX

HA (200 mg, 0.494 mmol of (β,1→4)-d-glucuronic acid-(β,1→3)-N-acetyl-d-glucosamine repeat unit, 1 eq.) was dissolved in 20 mL of MilliQ water. After complete HA dissolution, DMTMM·Cl (10.93 mg, 0.039 mmol, 0.08 eq.) was added to the solution and the mixture was stirred at room temperature (RT) for 2 h. Following this time, DOX (17.07 mg, 0.029 mmol, 0.06 eq.) was added to the mixture. The reaction was stirred at RT for 96 h and poured into 200 mL of cold ethanol (99%) to precipitate the HA-NH-DOX derivative. The final product was collected and desiccated under vacuum. The precipitate was dissolved in water, dialyzed against water to eliminate unreacted doxorubicin, and finally freeze-dried.

Yield: 180 mg (%*w*/*w*: 90%).

#### 2.2.2. General Protocol for the Synthesis of HA Hydrazides

HA (200 mg, 0.494 mmol, 1 eq.) was dissolved in 20 mL of MilliQ water. After total HA dissolution, DMTMM·Cl (10.93 mg, 0.039 mmol, 0.08 eq.) was added to the solution and the mixture was stirred at RT for 1 h. Following this time, the hydrazine derivative linker (0.06 eq.) was added to the mixture. The reaction was stirred at RT for 96 h and poured into 200 mL of cold ethanol (99%) to obtain the HA hydrazide derivative. The final product was collected and desiccated under vacuum. The precipitate was dissolved in water, dialyzed against water to eliminate unreacted amino-hydrazine derivative, and finally freeze-dried.

Yield(s): HA Adipic Hydrazide: 172 mg (%*w*/*w*: 86%); HA Hydrazide: 178 mg (%*w*/*w* 89%).

#### 2.2.3. General Protocol for the Synthesis of HA-Hydrazone-DOX

HA hydrazide (140 mg, 1 eq.) was dissolved in 20 mL of MilliQ water. After total conjugate dissolution, DOX (0.06 eq.) was added to the mixture and the pH of the solution was decreased to 5 by adding acetic acid. The mixture was stirred for 72 h at RT and poured into 200 mL of cold ethanol (99%) to obtain the HA-hydrazone-DOX derivative. The final product was collected and desiccated under vacuum.

Yield(s): HA-Adipic Hydrazone-DOX (HA-AdpHz-DOX): 104 mg (%*w*/*w*: 74%); HA-Hydrazone-DOX (HA-Hz-DOX): 92 mg (%*w*/*w*: 65%).

### 2.3. Characterization of HA-DOX Conjugates

#### 2.3.1. Nuclear Magnetic Resonance (NMR) 

First, 1H NMR spectra were recorded on a Bruker Ultrashield Advance II 400 MHz instrument. Samples were freshly prepared at a concentration of 5 mg/mL, using deuterated water as a solvent.

#### 2.3.2. Drug Loading

DOX loading was determined by UV–VIS at 488 nm using a calibration curve previously produced with DOX standard solutions in the concentration range of 1–200 μg/mL (R^2^ = 0.99). 

#### 2.3.3. Free Drug Content

The free DOX in the HA-DOX was quantified by high-performance liquid chromatography (HPLC). Conjugates were dissolved in PBS (2.0 mg/mL) and purified by vivaspin (15,000 rpm, 15 min, cut-off 10 kDa). The filtrated solution was analyzed using a Shimadzu Prominence system (Shimadzu, Kyoto, Japan). A nucleosilC18 (Macherey-Nagel, Duren, Germany) (150 × 4.6 mm; particle size 5 μm) column was used to separate the desired product. Then, 0.1% of formic acid in acetonitrile and 0.1% of formic acid in water (10%, 0 min; 90%, 13–15 min; 10%, 15–20 min) were used as mobile phases and applied in gradient mode. The quantification method was set with a detection wavelength of 480 nm, a sample injection volume of 10 μL, and a flow rate of 0.6 mL/min. Free DOX content was assessed by means of a previously performed DOX calibration curve (limit of detection (LOD) 2.4 µg/mL). 

#### 2.3.4. Dynamic Light Scattering (DLS)

The HA-DOX conjugates’ size was measured by using 0.5 mg/mL HA-DOX derivative in 10 mM phosphate, 150 mM NaCl, pH 7.4 (PBS). Measurements were performed using a Malvern Dynamic Light Scattering Zetasizer ZS (Malvern, Cambridge, UK) equipped with a red laser (λ = 633 nm) at a fixed angle of 173° at 25 °C. DLS measurements were performed in triplicate.

#### 2.3.5. Zeta Potential Analysis

The zeta potential was obtained using 0.5 mg/mL HA-DOX derivative in 1 mM KCl. Measurements were performed using a Malvern Dynamic Light Scattering Zetasizer ZS (Malvern, UK). Analyses were performed in triplicate.

### 2.4. Cell Lines

The murine glioma GL261 cells (DSMZ, German Collection of Microorganisms and Cell Cultures GmbH, Leibniz institute (Braunschweig, Germany) were grown at 37 °C, in a 10% CO_2_ atmosphere, and maintained in Dulbecco’s Modified Eagle Medium (DMEM) with l-Glutamine, 1 g/L d-Glucose, and sodium pyruvate. 

The human brain glioblastoma astrocytoma U87-MG cells and the murine melanoma B16F10 cells (American Type Culture Collection (ATCC, Manassas, VA, USA)) were grown at 37 °C, in a 5% CO_2_ atmosphere. Both cell lines were maintained in Minimum Essential Medium (MEM) without l-Glutamine and Eagle’s Minimal Essential Medium with L-Glutamine, respectively. 

All the media were supplemented with 10% fetal bovine serum (FBS) and 1.0% Penicillin/Streptomycin.

### 2.5. In Vitro Cell Viability

#### 2.5.1. In Vitro Cell Viability on 2D Models

GL261, U87-MG, and B16F10 cells (5 × 10^3^ cells/well), were seeded in 96-well plates. After 24 h, the cells were treated with free DOX, HA, HA-NH-DOX, HA-Hz-DOX, and HA-AdpHz-DOX conjugates, in the range of 0.0001–5 µM DOX equivalent concentration. After 72 h, cells were fixed with PFA (4% *v*/*v* in water) for 20 min and then were treated with 50 µL of crystal violet stain and incubated at room temperature for 30 min. The stain was washed three times with demineralized water and the plates left to dry overnight. Finally, cells were treated with a solubilization solution of methanol, and the crystal violet absorbance (λ: 560 nm) was read with the SpectraMax ID5 microplate reader (Molecular Devices; San Jose, CA, USA). Data were normalized with the untreated group (100% viability) and Triton-X 100 group (0% viability). IC_50_ values were calculated using a non-linear regression (curve-fit) mode.

#### 2.5.2. In Vitro Cell Viability on 3D Models

GL261 (1 × 10^3^ cells/well) cells were seed in U-shaped 96-well plates for the formation of the 3D culture in methyl cellulose. Methyl-cellulose-containing medium was prepared by dissolving 1.2% of methyl cellulose in complete DMEM, and 3D cells were maintained in complete growth medium supplemented by 20% of methyl-cellulose-containing medium. After 96 h, the spheroids were treated with DOX, HA, HA-NH-DOX, HA-Hz-DOX, and HA-AdpHz-DOX conjugates, in the range of 0.0001–10 µM (DOX equivalent concentration). Images of the spheroids were collected every 48–72 h per 14 days using the EVOS XL Core Imaging System (Thermo Fisher Scientific, CA, USA) and the area was measured using ImageJ. After 14 days, 20 µL per well of Alamar blue solution was added. The cells were incubated at 37 °C, 10% CO_2_, for 18 h and the fluorescence (λ_em_: 580 nm, λ_ex_: 620 nm) was measured using the SpectraMax ID5 microplate reader (Molecular Devices; San Jose, CA, USA). Data were normalized with the untreated group (100% viability) and Triton-X 100 group (0% viability). IC_50_ values were calculated using a non-linear regression (curve-fit) mode.

### 2.6. Cell Uptake and Trafficking

The cell uptake of HA-NH-DOX, HA-Hz-DOX, and HA-AdpHz-DOX conjugates compared with the free DOX was detected by fluorescence-activated cell sorting (FACS) and confocal microscopy analysis using the intrinsic fluorescence of DOX (λ_ex_: 488 nm).

#### 2.6.1. Flow Cytometry

GL261 (1 × 10^5^ cells/well) cells were seeded in a 24-well plate and maintained in complete growth medium. After 24 h of incubation, cells were treated with the DOX, HA-NH-DOX, HA-Hz-DOX, and HA-AdpHz-DOX conjugates (1 µM DOX equivalent concentration), respectively. In the case of the HA competition assay, GL261 cells were pre-treated with HA (5 µM) for 3 h, at 37 °C. After 1, 3, and 6 h of incubation, the cells were harvested with trypsin solution, suspended in complete growth medium, and centrifuged at 300× *g* at 4 °C for 5 min. The supernatant was removed, and cells were washed three times with PBS solution supplemented by 5 mg/mL of BSA and 100 µL of EDTA 0.5 M. Then, cells were transferred into a FACS 96-V-well plate for the flow cytometry analysis. Flow cytometry analysis was performed using the BD FACSVerse™ Cell Analyzer (BD Bioscience, Franklin Lakes, NJ, USA) in the FITC channel.

#### 2.6.2. Confocal Microscopy

Cell uptake: GL261 (5 × 10^4^ cells/well) cells were seeded in a 24-well plate and maintained in complete growth medium. After 24 h of incubation, cells were treated as described in Section 2.6.1. After 1 and 6 h of incubation at 37 °C, the medium was removed, and the cells were washed with PBS and fixed using PFA 2% *v*/*v* for 20 min at 4 °C. The staining of the nuclei was performed using 300 nM of DAPI solution in PBS for 15 min. After washing, the cover glasses were mounted in medium for fluorescence evaluation on the LSM800 inverted microscope (ZEISS, Oberkochen, Germany). Fluorescent signal intensity was measured and normalized with the area containing cells using the ImageJ software for three representative images.

Intracellular trafficking in lysosomes: GL261 (5 × 10^4^ cell/well) cells were treated as described above. After 6 h of incubation at 37 °C, the medium was removed, and the cells were permeabilized using 0.25% *v*/*v* of Triton 100-X in PBS solution for 10 min on ice. Following this time, lysosomes were stained with anti-mouse CD107a (LAMP-1) antibody and diluted 1:100 in PBS solution supplemented by 2% of BSA, for 1 h at 4 °C. Nuclei were stained using 300 nM DAPI solution in PBS for 15 min and analyzed. The colocalization analysis between DOX and LAMP-1 signals was performed using the “Coloc2” plugin in the ImageJ software and selecting the “Pearson’s R value (no threshold)” coefficient of three representative images.

### 2.7. In Vivo Study

All experiments were performed following the Belgian national regulation guidelines, as well as in accordance with EU Directive 2010/63/EU, and were approved by the ethical committee for animal care of the Faculty of Medicine of the Université Catholique de Louvain (2019/UCL/MD/004). Animals had free access to water and food. Animal bodyweight was constantly monitored throughout the experiments.

#### Anticancer Activity on GBM Tumor Model

The in vivo screening of the HA-NH-DOX, HA-Hz-DOX, and HA-AdpHz-DOX conjugates was performed on C57BL/6J mice (n = 5). GL261 (1.5 × 10^6^ cell/mouse) cells were subcutaneously implanted. After ten days, mice were randomized into 5 groups (5 mice/group) with an average tumor size of 35 mm^3^ and each group was intratumorally injected with PBS, DOX, HA-NH-DOX, HA-Hz-DOX, or HA-AdpHz-DOX (3 mg/kg in DOX equivalent, 35 µL). Tumor growth measurement was assessed every two days by measuring the tumor volumes with a caliper and according to the following formula:

$$V=\frac{x\times y\times z\times \pi }{6}$$

Mice where sacrificed according to the following endpoints: (i) the tumor volumes reached 800 mm^3^; (ii) mice lost 20% of their bodyweight, or (iii) 10% bodyweight loss plus clinical signs of distress (paralysis, arched back, lack of movement).

### 2.8. Statistical Analysis

Statistical analysis was performed using GraphPad Prism, version 7.0a (GraphPad Software, San Diego, CA, USA). All results are expressed as mean ± standard deviation (SD), except for results arising from in vivo studies, which are expressed as mean ± standard error of the mean (SEM). The half maximal inhibitory concentration (IC_50_) values were calculated using a nonlinear regression log(inhibitor) vs. response, variable slope. Statistical significance was attained for values of *p* < 0.05 and determined using one-way ANOVA for the in vitro and in vivo studies (tumor growth vs. time). Survival curves were compared using a Mantel–Cox (log-rank) test. Outliers were calculated using GraphPad software (significance level 0.01, two-sided) and removed from the study.

## 3. Results and Discussion

### 3.1. Synthesis and Characterization of HA-DOX Conjugates

The administration of simple drugs suffers from limitations including (1) clearance from the brain, reducing the effective drug concentration; (2) metabolic activity that can alter the active molecular structure by switching towards inactive or toxic metabolites; (3) no sustained release. An attractive strategy resides in conjugation to HA, which can overcome drug limitations and lead to superior therapeutic outcomes.

**Scheme 1:**
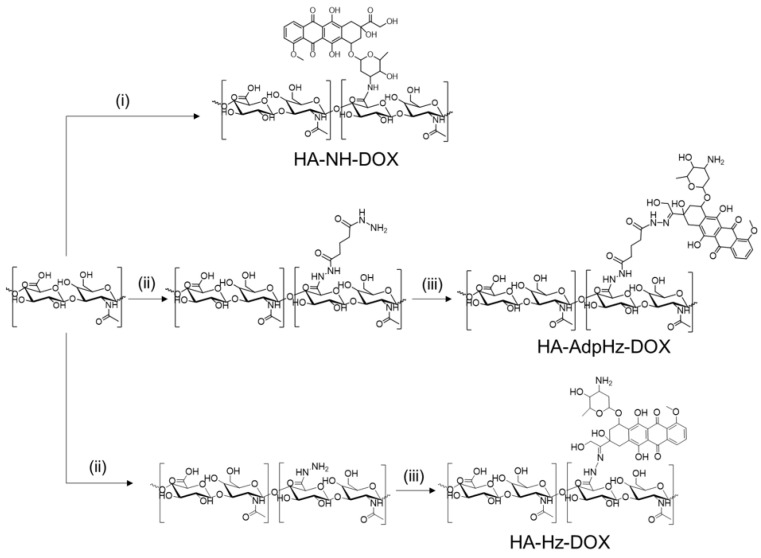
Synthetic scheme followed to achieve hyaluronic acid–doxorubicin (HA-DOX) conjugates. (**i**) DMTMM Cl, water, 2 h, RT; DOX, water, 96 h. (**ii**) DMTMM Cl, water, 2 h, RT; hydrazine linker, water, 96 h. (**iii**) DOX, CH_3_COOH (pH = 5), water, 96 h.

DOX was conjugated to HA (100 kDa) by means of three different linking chemistries: (1) direct conjugation via DMTMM chemistry, forming an amide bond and (2) through a flexible, hydrophobic, and long adipic dihydrazide or (3) short hydrazide linker (see Scheme 1). In the latter two strategies, the linking chemistry allowed the generation of a pH-responsive hydrazine, possibly through 13 DOX ketone [32,33].

HA-DOX conjugates were characterized in terms of drug loading, size, zeta potential, and free drug (Table 1). The HA conjugates synthesized in this study showed comparable drug loading while maintaining conjugate solubility, with no visible aggregates. The negligible presence of unbound DOX was detected. ^1^H NMR characterization of the synthesized HA derivatives was used to determine their identity and purity. In the representative NMR spectra of conjugates, the covalent incorporation of DOX into the HA polymer backbone resulted in the expected widening of the characteristic drug signals (Figure S1). However, the quantification of DOX loading by NMR was not possible due to the partial overlapping and shielding of protons in the aromatic regions due to micellization [26]. After conjugating HA to DOX, DLS analysis showed the formation of small, self-assembled structures with a size between 5 and 25 nm. It is worth noting that the linking chemistry had a role in the size: HA-NH-DOX conjugates had the smallest size (average of 6.9 nm), while HA-Hz-DOX and HA-AdpHz-DOX showed an increase in size (15.9 nm and 25.2 nm, respectively) (Figure S2). We speculate that the amide bond induced the formation of bulky structures, while the conjugation of DOX with a linker promoted the assembly of relatively larger nanoaggregates. Brain tumors possess substantially higher cell density and greater collagen content than normal brain tissue, thus preventing the adequate diffusion of the nanocarrier within the GBM microenvironment [13,14]. The size of the constructs should allow infiltrative cells to be reached that cause tumor recurrence and to provide more uniform drug distribution within tumors and diffusion within the brain [13].

### 3.2. HA-DOX Conjugates Show Superior Cytotoxicity to Free Drug in Both 2D and 3D Models

**Figure 1:**
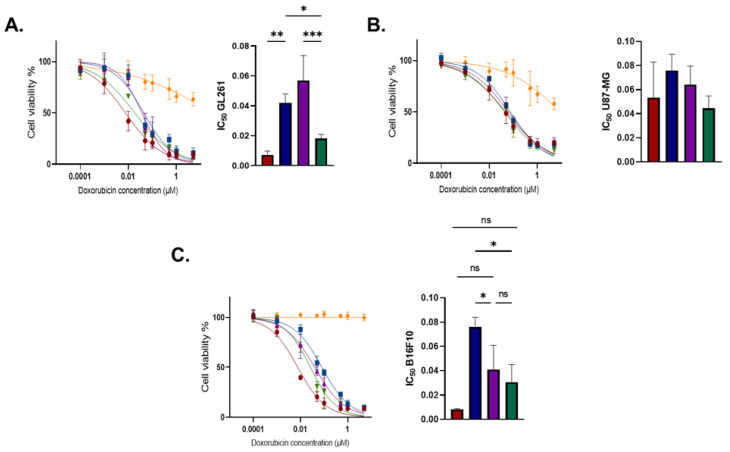
Effect of DOX and HA-DOX conjugates on cell viability. (**A**) GL261, (**B**) U87-MG, and (**C**) B16F10 were incubated with free DOX, HA, HA-NH-DOX, HA-AdpHz-DOX, and HA-Hz-DOX conjugates, in a concentration range of 0.0001–5 µM in DOX equivalent. After 72 h of incubation, the cell viability assay was performed via crystal violet staining, and data were obtained by calculating the crystal violet mean fluorescence intensity. Mean, error bar = SD, N = 3, statistical analysis obtained by one-way ANOVA multiple comparison (ns = not significant, * *p* < 0.05, ** *p* < 0.01, *** *p* < 0.001). Free DOX: red; HA: yellow; HA-NH-DOX: blue, HA-AdpHz-DOX: violet; HA-Hz-DOX: green.

Cell viability was measured after exposure to HA conjugates and free DOX. Therefore, DOX, HA, and HA-DOX were tested on three tumor cell lines, murine glioblastoma cells GL261, human glioblastoma cells U87-MG, and murine melanoma B16F10, after 72 h exposure [34]. Figure 1 reports the cell viability profiles of GL261, U87-MG, and B16F10 incubated with the drug conjugates.

The linking chemistry drastically altered the in vitro cytotoxic effects, in agreement with other reports. DOX needs to be released after internalization and must localize into the nuclei of cancer cells for DNA intercalation [35]. The conjugation of DOX to HA with Hz led to higher efficacy when compared to the amide bond linker. HA-NH-DOX possesses a stable bond and DOX release will happen as a consequence of the HA backbone’s degradation into the cells, while HA-Hz-DOX and HA-AdpHz-DOX contain two hydrazone linkers of different length, which allow DOX release in acidic conditions. Concerning GL261 (Figure 1A), the IC_50_ obtained with DOX was lower than that obtained with the HA-NH-DOX and HA-AdpHz-DOX conjugates. Indeed, usually, DOX bioconjugates possess significantly lower cytotoxicity compared to the free drug, which implies the use of high drug doses [33,36]. This is particularly critical in the context of GBM, where, for anatomical unicity (BBB, neurons, etc.), the use of large amounts of drugs is usually precluded [18]. HA-Hz-DOX showed superior activity compared to the HA-AdpHz-DOX and HA-NH-DOX conjugates and no significant difference compared to free DOX. This can be ascribed to the more efficient/rapid DOX release from the HA backbone, allowing the quicker onset of free DOX into the cells, and therefore higher efficacy. In U87-MG cells, no statistical difference in toxicity between HA-DOX and DOX was observed (Figure 1B). The higher IC_50_ than in GL261 might be related to several factors, such as (i) cell line sensitivity to the drug, rapid proliferation, and aggressiveness; (ii) different intracellular trafficking and intracellular release rate. Finally, the efficacy of the novel compounds on B16F10 was explored (Figure 1C). We selected this cell line due to the high rate of metastasis of melanoma towards the brain [37]. HA-Hz-DOX had a lower IC_50_ compared to HA-AdpHz-DOX and HA-NH-DOX, in agreement with GL261 cells. Interestingly, HA showed slight cytotoxicity towards GL261 and U87MG in a concentration-dependent manner, suggesting that HA with a MW of 100 kDa could play an active role towards GBM cells, in agreement with previous reports [38]. However, no effect was observed on B16F10. We can speculate that the degradation of HA could activate signaling pathways in GBM, leading to cell death, which did not occur in the melanoma cells [38,39,40]. Taken together, these results support the hypothesis that HA can ameliorate the efficacy of the drug DOX by providing targeting to GBM cells expressing an activated form of CD44 (see Section 4). Since HA-DOX showed higher performance in GL261, we decided to focus our study on this cell line.

**Figure 2:**
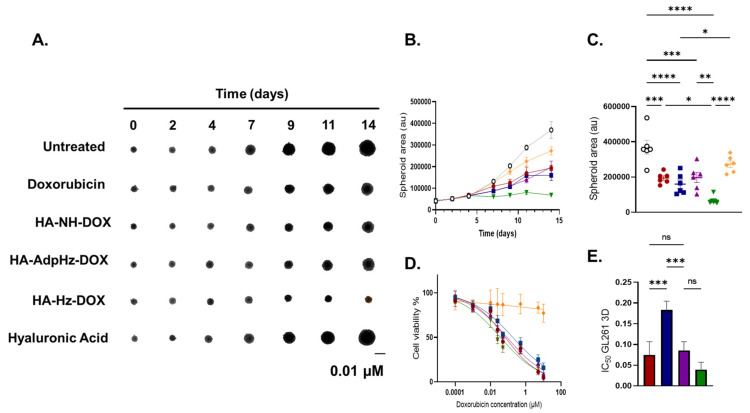
Effect of DOX and HA-DOX conjugates on 3D GL261 spheroids. (**A**) Representative images of 3D cell growth 0–14 days after treatment with 0.01 µM in DOX equivalent of free DOX, HA, HA-NH-DOX, HA-AdpHz-DOX, and HA-Hz-DOX conjugates; pictures were obtained by using EVOS XL Core Imaging System and processed by ImageJ. (**B**) Three-dimensional cell growth after treatment with free DOX, HA, HA-NH-DOX, HA-AdpHz-DOX, and HA-Hz-DOX conjugates in the range of 0–14 days (0.01 µM in DOX equivalent). (**C**) Distribution of spheroid area at day 14. (**D**) Three-dimensional cell viability after 15 days of treatment with free DOX, HA, HA-NH-DOX, HA-AdpHz-DOX, and HA-Hz-DOX conjugates in the concentration range of 0.0001–10 µM. Cell viability was obtained by reading the Alamar blue fluorescence (λ_em_: 580 nm, λ_ex_: 620 nm) and processed through GraphPad (Mean, error bar = SD, N = 4). (**E**) Free DOX, HA-NH-DOX, HA-AdpHz-DOX, and HA-Hz-DOX conjugates’ IC_50_. Data were obtained by cell viability assay on GL261 3D culture and processed through GraphPad (Mean, error bar = SD, N = 4, statistical analysis obtained by one-way ANOVA multiple comparison: ns = not significant, * *p* < 0.05, ** *p* < 0.01, *** *p* < 0.001, **** *p* < 0.0001). Free DOX: red; HA: yellow; HA-NH-DOX: blue, HA-AdpHz-DOX: violet; HA-Hz-DOX: green.

Compared to simplified two-dimensional (2D) in vitro GBM cultures, spheroids possess a 3D architecture, allowing the study of nanocarriers’ interaction with both the external and inner core, mimicking more closely the physiological context in comparison to other in vitro models. Additional advantages reside in the possibility to maintain spheroid cultures for a long time, thus allowing us to understand the impact of long-term treatments [41]. The anticancer activity of HA-DOX was explored on in vitro 3D GL261 spheroids by measuring the area as a function of time and by Alamar blue assay following cells’ treatment with equivalent DOX concentrations in the range of 0.0001–10 µM for 14 days. Figure 2 reports the GL261 spheroid area measured every 2–3 days during the 14 days of treatment.

All the treatments showed higher efficacy compared to the untreated group. GL261 spheroids treated with HA-Hz-DOX demonstrated higher growth inhibition and the spheroid area was mostly the same throughout the time period. Spheroids treated with the HA-NH-DOX and HA-AdpHz-DOX conjugates displayed similar behavior as DOX, where the spheroid area constantly grew throughout the time period, without significant differences in terms of area, while HA-NH-DOX showed lower cytotoxicity than HA-AdpHz-DOX and free DOX. These differences in behavior can be explained by the linking chemistry. The GBM microenvironment is characterized by an acidic pH (6–6.8). Some release of DOX in HA-AdpHz-DOX conjugates could occur prior to uptake, while HA-Hz-DOX has a bulkier conformation and could allow release only after cell internalization in the lysosome compartment (pH ~5.5) [33,42]. On the other hand, HA-NH-DOX is a pH-stable linker, preventing release in physiological conditions; we expect DOX release from HA to occur by hyaluronidase-driven degradation [43]. Figure 2D,E show the IC_50_ obtained after spheroid treatments with DOX and HA-DOX conjugates. HA-Hz-DOX showed slightly lower IC_50_ to DOX (even if without statistical significance). Contrary to what was observed in the 2D model, HA-AdpHz-DOX showed similar cytotoxicity to DOX, while HA-NH-DOX possessed a higher IC_50_. Moreover, spheroids treated with HA showed slight tumor growth inhibition, in agreement with the results observed in the 2D model, suggesting that HA could interfere with the process of tumor growth. By comparing 2D and 3D models, a similar trend in terms of the cytotoxic activity of HA-DOX conjugates could be observed. In particular, HA-Hz-DOX showed the better activity in both models and did not show a significant difference from DOX in terms of IC_50_. This could be attributed to the combination of the high cell internalization and intracellular DOX release from the HA backbone by means of the hydrazone linker (see Section 3.3). HA-AdpHz-DOX showed better efficacy on 3D models than 2D. 

### 3.3. HA-DOX Conjugates Facilitate DOX Internalization and Promote Chemotherapeutic Efficacy

**Figure 3:**
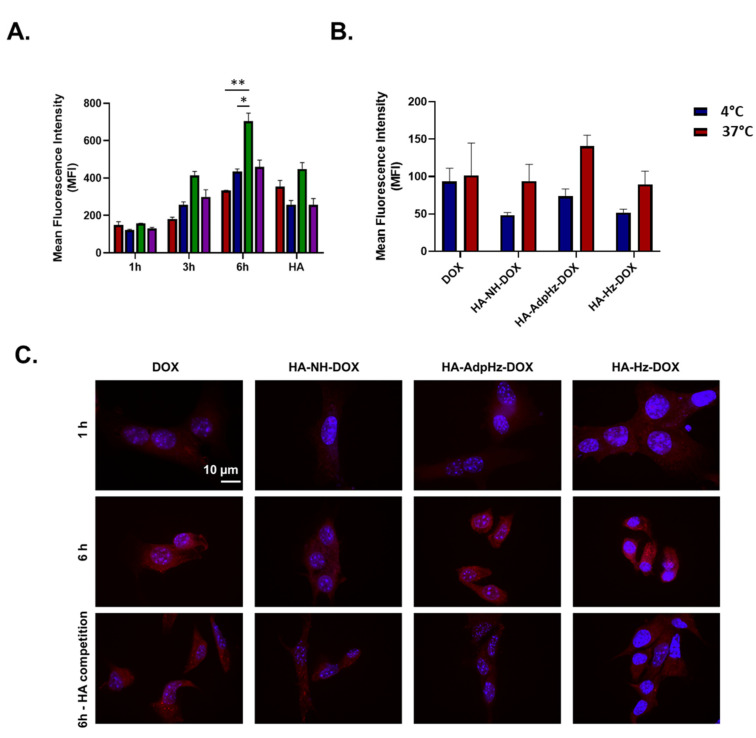
GL261 cell uptake of DOX and HA-DOX conjugates. (**A**) Cell uptake of DOX equivalent concentration of free DOX, HA-NH-DOX, HA-AdpHz-DOX, and HA-Hz-DOX conjugates (1 µM in DOX equivalent concentration) at 1, 3, and 6 h by FACS analysis. HA competition assay was assessed by pre-incubating the cells with free HA (5 µM) (Mean, error bar = SD, N = 3). (**B**) FACS analysis on GL261 cell uptake at 4 °C and 37 °C. (**C**) Representative confocal microscopy images of GL261 cell uptake of DOX equivalent concentration of free DOX, HA-NH-DOX, HA-AdpHz-DOX, and HA-Hz-DOX conjugates (1 µM in DOX equivalent concentration) at 1 and 6 h. HA competition assay was assessed by pre-incubating the cells with free HA (5 µM). Nuclei were stained with DAPI (DAPI: blue; DOX: red). Data were processed through GraphPad (Mean, error bar = SD, N = 3, statistical analysis obtained by one-way ANOVA multiple comparison: ns = not significant, * *p* < 0.05, ** *p* < 0.01). Free DOX: red; HA: yellow; HA-NH-DOX: blue, HA-AdpHz-DOX: violet; HA-Hz-DOX: green.

Taking advantage of the intrinsic fluorescence of DOX, the intracellular uptake of DOX and HA-DOX conjugates in GL261 was investigated by FACS analysis and immunofluorescence confocal microscopy. The quantitative cell uptake data obtained by FACS analysis of GL261 cells incubated with the DOX HA-NH-DOX, HA-AdpHz-DOX, and HA-Hz-DOX conjugates, reported in Figure 3A, show a progressive increase in DOX-associated fluorescence, demonstrating that HA-DOX conjugates were taken up by cells in a time-dependent manner. All the conjugates showed higher uptake compared to the free DOX. In particular, after 3 h of incubation, HA-Hz-DOX conjugates showed higher internalization compared to the other conjugates analyzed and the free DOX. The higher internalization was observed after 6 h. We hypothesize that this trend can be explained by the presence of the CD44 receptor on the GL261 surface. Therefore, the presence of the CD44 receptor on the GL261 surface was demonstrated (Figure S3). CD44 is a cell membrane glycoprotein that is overexpressed in GBM cells and promotes the internalization of the cells, promoting cell motility, proliferation, apoptosis, and angiogenesis, which, when pathologic, are characteristic of malignancy. To prove the implication of CD44 in the HA-DOX uptake, an HA competition study was performed. A strong decrease in the cell uptake of HA-DOX after free HA pre-treatment was observed after 3 h, while this pre-treatment did not alter the uptake of free DOX (Figure 3A). These results were supported by cell uptake inhibition at 4 °C (Figure 3B), confirming the energy-driven mechanism of cell internalization. 

Figure 3C shows representative images of GL261 cells incubated with DOX, HA-NH-DOX, HA-AdpHz-DOX, and HA-Hz-DOX for 1 and 6 h at 37 °C and the competition assay with HA. After 1 h of incubation, no difference in fluorescence was observed among the free DOX and the conjugates. After 6 h, high DOX accumulation in the nuclei was found, resulting in the released DOX’s localization in the nuclei. Fluorescence quantification of the signals is reported in Figure S4.

**Figure 4:**
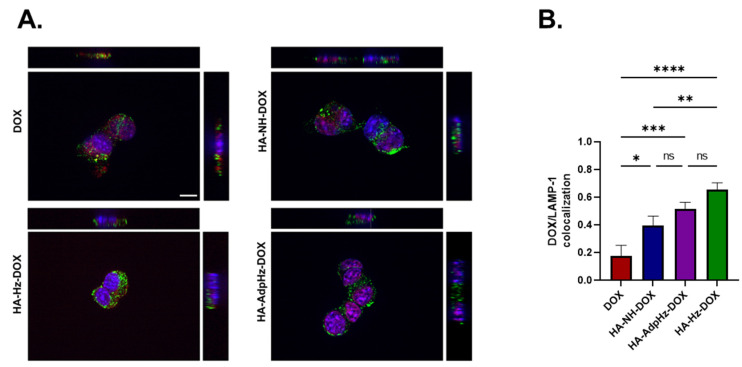
Confocal microscopy images of GL261 cells for lysosome accumulation assay. (**A**) GL261 cells were incubated with free DOX, HA-NH-DOX, HA-AdpHz-DOX, and HA-Hz-DOX conjugates for 6 h, nuclei were stained with DAPI, and lysosomes were stained with the LAMP-1 antibody. Pictures were obtained by Z-stack imaging (DAPI: blue; DOX: red; LAMP-1 antibody: green; LAMP-1+DOX: yellow). (**B**) Quantification of colocalization analysis for DOX or HA-DOX conjugates with LAMP-1 (Pearson’s coefficient) (Mean, error bar = SD, N = 3, statistical analysis obtained by one-way ANOVA multiple comparison: ns = not significant, * *p* < 0.05, ** *p* < 0.01, *** *p* < 0.001, **** *p* < 0.0001).

The colocalization of free or conjugated DOX (red fluorescence) within lysosomes (green fluorescence) was investigated by labeling lysosomes with LAMP-1 antibody. Representative Z-stack images of GL261 cells treated with DOX, and HA-DOX conjugates, show the overlap between the fluorescence of the LAMP-1 antibody and the DOX fluorescence (Figure 4). Yellow spots resulting from the overlapping of red conjugates colocalized with the lysosome compartment’s green staining were observed in samples treated with the HA-Hz-DOX and HA-NH-DOX conjugates, confirming the accumulation inside the lysosomes, crucial for DOX release, as demonstrated by Pearson’s coefficient analysis (Figure 4B). However, cells incubated with free DOX and HA-AdpHz-DOX displayed a low LAMP-1 antibody and DOX fluorescence overlap: both seemed to be dispersed in the cytoplasm and in the nuclei of the cells. This behavior suggests that HA-AdpHz-DOX releases DOX more rapidly, probably in the extracellular milieu, and therefore does not accumulate inside the lysosomes, in agreement with the cytotoxic results described in Section 2. The DOX fluorescence associated with HA-Hz-DOX was mainly found in the lysosomes or in the cytosol. These results confirm the intracellular delivery of the drug by the HA and the lysosomal trafficking, where the presence of the acidic environment can trigger the cleavage of the pH-cleavable hydrazone bond, promoting the drug release. However, the brain possesses a complex structure with interconnected factors such as pH, volume of interstitial fluid, and mechanical properties, which vary depending on the region and on the disease (cancer stage and dimensions, injury deriving from resections, etc.). In addition, the exchange in the brain is regulated through several bio-compartments, including the blood, the extracellular fluids (ECF), the cerebrospinal fluid (CSF), and the cells (healthy and not). The in vitro study of the behavior of the conjugates cannot replicate the brain architecture, although it is clear that the linking chemistry strongly influences the efficacy.

### 3.4. Anticancer Activity on GBM Tumor Model

**Figure 5:**
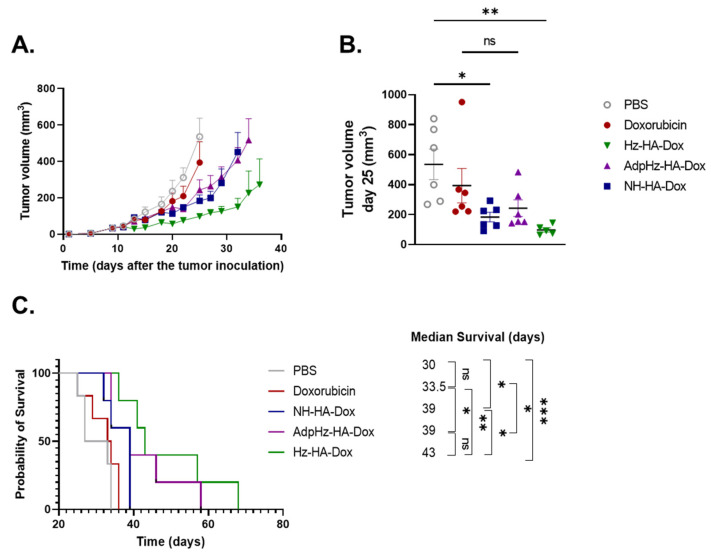
Anti-tumor effect on GL261 tumor-bearing mice model treated with DOX and HA-DOX. (**A**) C57BL/6J mice were treated with PBS, DOX, HA-NH-DOX, HA-Hz-DOX, and HA-AdpHz-DOX conjugates (3 mg/kg in DOX equivalent) after 10 days following tumor transplantation. Tumor growth was delayed in the HA-Hz-DOX group (green); in the PBS (grey) and the DOX (red) groups, the tumor growth was higher in comparison to the groups treated with the HA-DOX conjugates; in the HA-NH-DOX (blue) and the HA-AdpHz-DOX (violet) groups, the tumor growth rate was overlapped. Each point represents the mean of tumor size. (**B**) Comparison of tumor distribution at day 25, indicating that HA-Hz-DOX was statistically significant compared to untreated and DOX. (**C**) Kaplan–Meier curves: HA-Hz-DOX and HA-AdpHz-DOX groups presented the highest survival rate. (Mean, error bar = SEM, N = 5, statistical analysis obtained by one-way ANOVA multiple comparison: ns = not significant, * *p* < 0.05, ** *p* < 0.01, *** *p* < 0.001).

The in vivo anti-tumor efficacy of HA-DOX conjugates was assessed on subcutaneous GL261-bearing mice (Figure 5). GL261 cells were ectopically transplanted and intratumorally treated with the DOX, HA-NH-DOX, HA-Hz-DOX, and HA-AdpHz-DOX conjugates. We selected this administration route since the local delivery of therapeutic agents at the tumor site is appealing for brain tumors (either by convention-enhanced delivery or filling the resection cavity), which are surrounded by a unique and protective tumor microenvironment, strongly limiting access for most chemotherapeutic agents. Moreover, this approach is highly translational since it can be easily implemented with the current standard of care.

The dose of treatment was optimized on the basis of previous reports [16,44]. All the HA-DOX constructs induced a delay in tumor growth compared to the PBS and DOX control groups. Moreover, similar tumor volumes were found between HA-NH-DOX and HA-AdpHz-DOX, while the tumor growth of HA-Hz-DOX displayed a slower growth rate compared to all the other groups. Importantly, no substantial change in bodyweight was registered throughout the experiment (Figure S5). The analysis of tumor size at day 25 (last day before the first sacrifice) showed no difference among the HA-DOX (Figure 5B); moreover, HA-Hz-DOX and HA-NH-DOX were significantly different compared to the PBS group (*p* < 0.01 and *p* < 0.05, respectively) but not with DOX. As shown in the Kaplan–Meier survival curves (Figure 5C), DOX slightly increased the mice’s survival but in a non-significant manner (33.5 days vs. 30 days for untreated mice), showing the low therapeutic effect of free DOX on this tumor model. The median survival time increased after administration of HA-DOX conjugates. Mice treated with HA-NH-DOX showed a similar median survival time to HA-AdpHz-DOX (39 days for both treatments). Mice treated with HA-Hz-DOX showed an enhanced median survival time (43 days) without a significant difference from HA-NH-DOX and HA-AdpHz-DOX (*p* < 0.05). However, the HA-Hz-DOX group displayed a significative difference compared to the free DOX (*p* < 0.01) and PBS (*p* < 0.001) groups. The differences in terms of survival can be explained by the strategic design of HA-DOX conjugates with a pH-cleavable bond (both Hz- and AdpHz) that allows faster drug onset compared to the enzymatic degradation of the polymeric chain, which will release the drug more slowly. This not only improves the therapeutic efficacy in terms of survival but, in the case of HA-Hz-DOX, induces slower tumor progression.

## 4. Conclusions

We report on the benefits of using HA-DOX conjugates as effective biodegradable nanomedicine in the local treatment of GBM. By conjugating DOX to HA, we demonstrate a more effective inhibition in tumor growth in an aggressive subcutaneous GBM tumor model compared to the free drug. Moreover, this study highlights the importance of understanding the design of bio-responsive linkers in the development of tailored conjugates for the local treatment of GBM in terms of drug release kinetics and brain diffusion, allowing the targeting of GBM cells, with an overall improvement in survival. This simple but effective polymer–drug conjugate displays high translational potential for future applications (i.e., intratumoral injection, convention-enhanced delivery, filling the tumor cavity) towards the advanced local treatment of GBM.

## Figures and Tables

**Table 1:** Characterization of HA-DOX conjugates.

Conjugate	DOX Loading (%*w*/*w*)	Size (nm)	Zeta Potential (mV)	Free Dox (%*w*/*w* of Total DOX)
HA-NH-DOX	6.12	6.9	−21.6	1.32
HA-AdpHz-DOX	5.72	25.2	−22.0	<LOD
HA-Hz-DOX	6.71	15.9	−21.4	<LOD

## Data Availability

Data are contained within the article and supplementary material.

## Supplementary Materials

The following supporting information can be downloaded at: https://www.mdpi.com/article/10.3390/pharmaceutics14010124/s1, Figure S1: Representative 1H NMR spectra (D2O, 400 MHz) of HA-DOX conjugates used in the study; Figure S2: Representative Dynamic Ligh Scattering profiles of (A) Hyaluronic acid; (B) HA-NH-DOX; (C) HA-AdpHz-DOX and (D) HA-Hz-DOX, Figure S3: Representative confocal images of GL261 stained with FITC anti-mouse/human CD44 Antibody, Figure S4: Quantification of confocal microscopy images of DOX signal from DOX and HA-DOX conjugates in GL261 in presence/absence of HA. Quantification was performed using the ImageJ programme from at least three images per treatment. Error bars represent the SD of the mean, Figure S5: Body weight change of mice after the treatments along the time.
