# Supplementary material for: Design of Bio-Responsive Hyaluronic Acid–Doxorubicin Conjugates for the Local Treatment of Glioblastoma
Source: Pharmaceutics. 2022 Jan 5;14(1):124. doi: 10.3390/pharmaceutics14010124 (PMC8781529; doi:10.3390/pharmaceutics14010124)
Supplement: Supplementary file 1 [file pharmaceutics-14-00124-s001.zip › pharmaceutics-1513845-supplementary.pdf]

pharmaceutics

Supplementary Materials: Design of Bio-Responsive Hyaluronic Acid-Doxorubicin Conjugates for the Local Treatment of Glioblastoma

Alessio Malfanti, Giuseppina Catania, Quentin Degros, Mingchao Wang, Mathilde Bausart, Véronique Préat

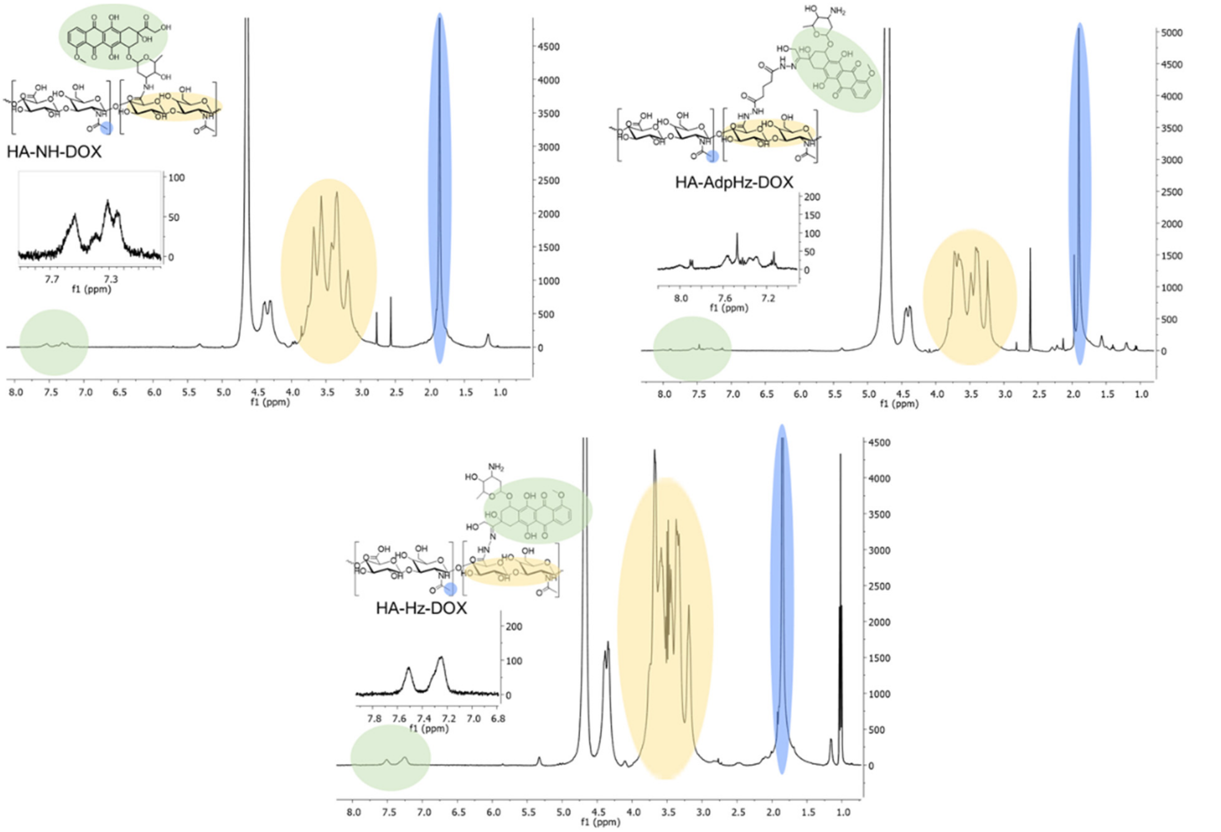

Figure S1. Representative 1H NMR spectra (D_2O, 400 MHz) of HA-DOX conjugates used in the study.

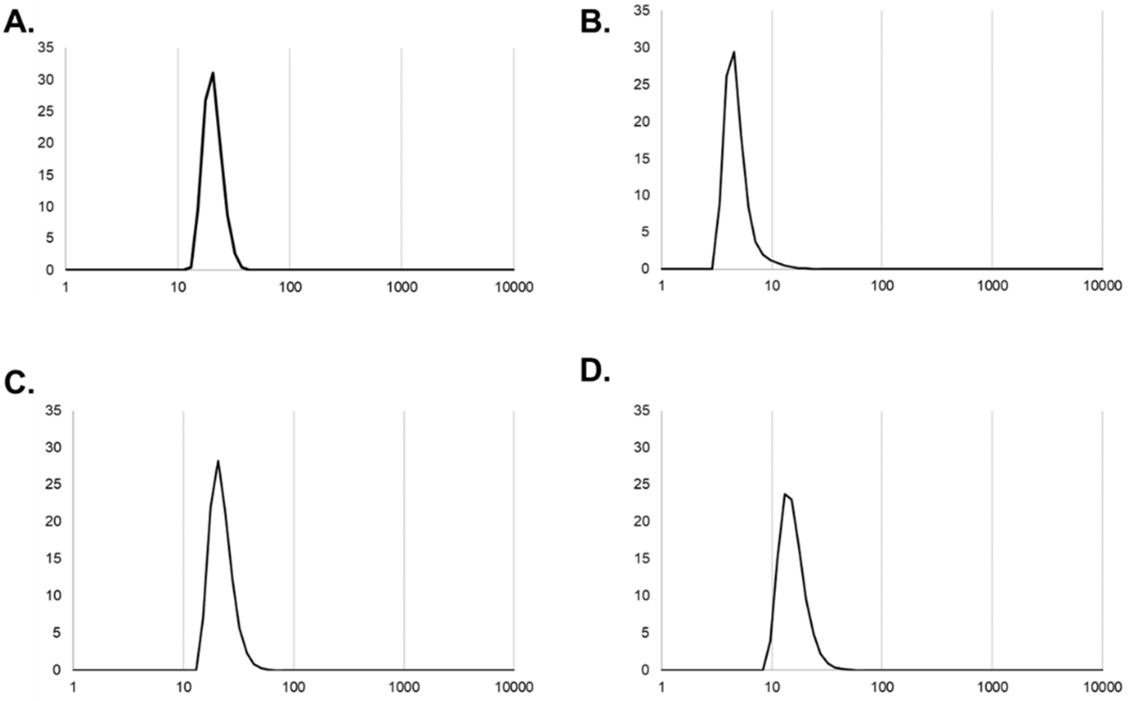

Figure S2. Representative Dynamic Ligh Scattering profiles of **A.** Hyaluronic acid; **B.** HA-NH-DOX; **C.** HA-AdpHz-DOX and **D.** HA-Hz-DOX.

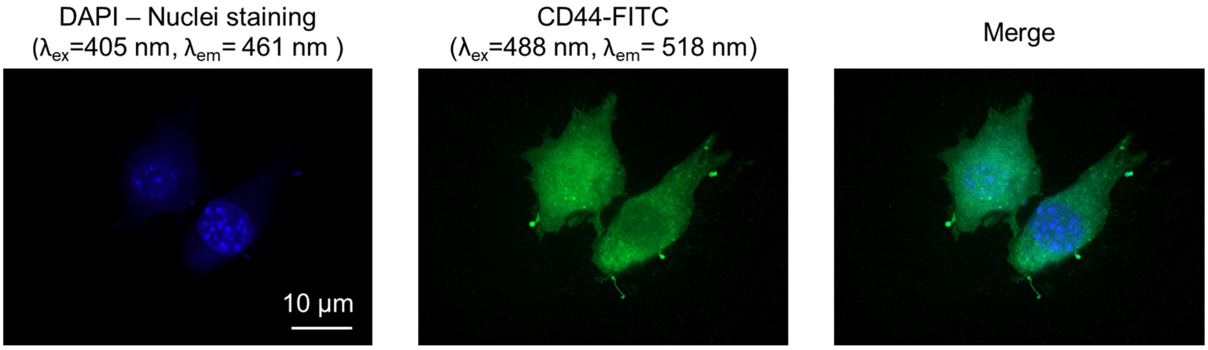

Figure S3. Representative confocal images of GL261 stained with FITC anti-mouse/human CD44 Antibody.

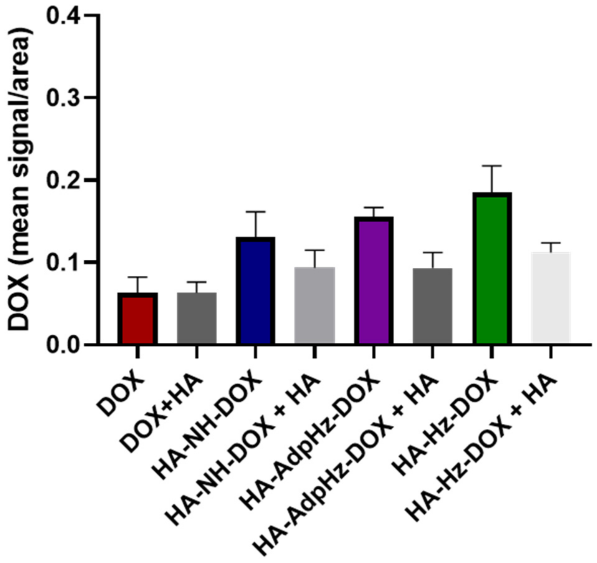

Figure S4. Quantification of confocal microscopy images of DOX signal from DOX and HA-DOX conjugates in GL261 in presence/absence of HA. Quantification was performed using the ImageJ programme from at least three images per treatment. Error bars represent the SD of the mean.

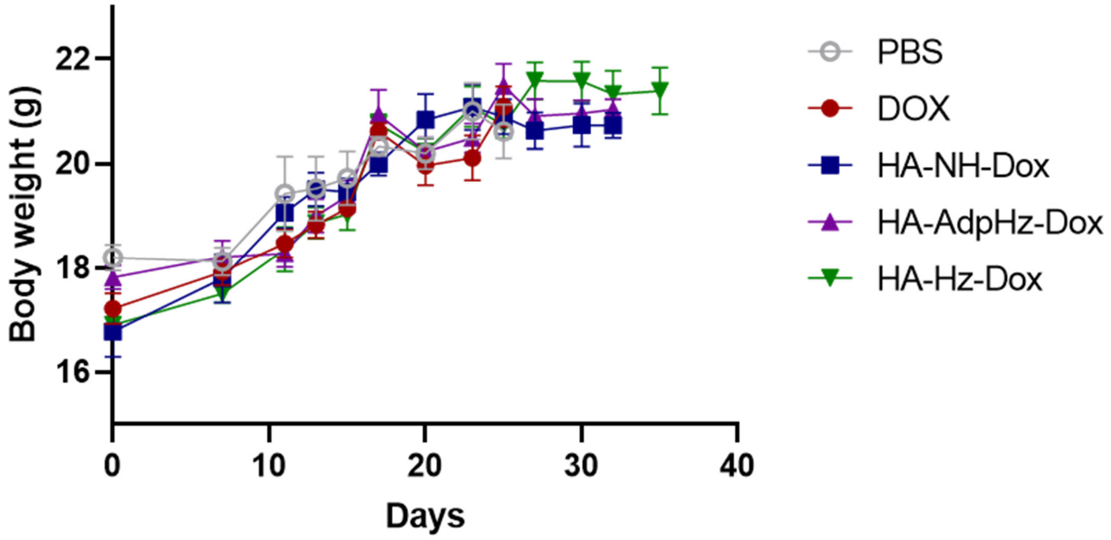

Figure S5. Body weight change of mice after the treatments along the time.
